# AutoScore: A Machine Learning–Based Automatic Clinical Score Generator and Its Application to Mortality Prediction Using Electronic Health Records

**DOI:** 10.2196/21798

**Published:** 2020-10-21

**Authors:** Feng Xie, Bibhas Chakraborty, Marcus Eng Hock Ong, Benjamin Alan Goldstein, Nan Liu

**Affiliations:** 1 Programme in Health Services and Systems Research Duke-NUS Medical School Singapore Singapore; 2 Department of Statistics and Applied Probability National University of Singapore Singapore Singapore; 3 Department of Biostatistics & Bioinformatics Duke University Durham, NC United States; 4 Department of Emergency Medicine Singapore General Hospital Singapore Singapore; 5 Health Services Research Centre Singapore Health Services Singapore Singapore; 6 Institute of Data Science National University of Singapore Singapore Singapore

**Keywords:** clinical decision making, machine learning, prognosis, clinical prediction rule, electronic health records

## Abstract

**Background:**

Risk scores can be useful in clinical risk stratification and accurate allocations of medical resources, helping health providers improve patient care. Point-based scores are more understandable and explainable than other complex models and are now widely used in clinical decision making. However, the development of the risk scoring model is nontrivial and has not yet been systematically presented, with few studies investigating methods of clinical score generation using electronic health records.

**Objective:**

This study aims to propose AutoScore, a machine learning–based automatic clinical score generator consisting of 6 modules for developing interpretable point-based scores. Future users can employ the AutoScore framework to create clinical scores effortlessly in various clinical applications.

**Methods:**

We proposed the AutoScore framework comprising 6 modules that included variable ranking, variable transformation, score derivation, model selection, score fine-tuning, and model evaluation. To demonstrate the performance of AutoScore, we used data from the Beth Israel Deaconess Medical Center to build a scoring model for mortality prediction and then compared the data with other baseline models using the receiver operating characteristic analysis. A software package in R 3.5.3 (R Foundation) was also developed to demonstrate the implementation of AutoScore.

**Results:**

Implemented on the data set with 44,918 individual admission episodes of intensive care, the AutoScore-created scoring models performed comparably well as other standard methods (ie, logistic regression, stepwise regression, least absolute shrinkage and selection operator, and random forest) in terms of predictive accuracy and model calibration but required fewer predictors and presented high interpretability and accessibility. The nine-variable, AutoScore-created, point-based scoring model achieved an area under the curve (AUC) of 0.780 (95% CI 0.764-0.798), whereas the model of logistic regression with 24 variables had an AUC of 0.778 (95% CI 0.760-0.795). Moreover, the AutoScore framework also drives the clinical research continuum and automation with its integration of all necessary modules.

**Conclusions:**

We developed an easy-to-use, machine learning–based automatic clinical score generator, AutoScore; systematically presented its structure; and demonstrated its superiority (predictive performance and interpretability) over other conventional methods using a benchmark database. AutoScore will emerge as a potential scoring tool in various medical applications.

## Introduction

Risk-scoring models are sparse models with integer point scores, which are used pervasively throughout medicine for risk stratification [1]. Risk-scoring models have been developed to determine which patients are at most risk of adverse events or worsening health conditions. Accurate identification of patients at risk can be useful for appropriate allocations of medical resources [2-4]. Risk-scoring models have been traditionally developed in 1 of 2 ways: through expert opinions or consensus, such as the Sepsis-related Organ Failure Assessment [5] score and the National Early Warning Score [6], and through the analysis of conventional cohort studies, such as the History, Electrocardiogram, Age, Risk factors, and Troponin score [7] and the Charlson Comorbidity Index [8]. Both approaches are labor-intensive and are not easy to update over time, which reveals the need for a flexible and fast approach to deriving risk-scoring models.

At present, the increasing popularity of electronic health records (EHRs) [9] creates an opportunity to take advantage of its growing quantity and diversity of data for creating novel risk models with both domain expert–curated approaches and advanced machine learning solutions. Although EHRs are rich data sources, numerous data items are collected in a nonsystematic way related to clinical use, leading to a bevy of irrelevant and redundant information. Therefore, variable selection, the process of determining a subset of relevant and discriminative variables for model development [10], plays an essential role in the development of a risk model. In risk models, more variables do not necessarily lead to better performance [11]. Moreover, irrelevant and redundant information can adversely affect model interpretability and accessibility, especially in the clinical context. A typical but time-intensive approach for variable selection uses domain knowledge obtained from literature reviews and consultation with experts; however, the literature may not always be available, and the expert’s interpretation could be biased. Analytic approaches exist, such as stepwise methods (eg, forward and backward) and regularization (eg, the least absolute shrinkage and selection operator [LASSO]). However, when data sets are large enough, these methods do not often achieve a sparse solution. Thus, there is an unmet need to develop a parsimonious model with easy access to validation in the context of EHRs.

Model complexity not only affects model efficiency but also impacts transparency and interpretability [12] in clinical practice. Although machine learning often has greater predictive accuracy than simpler models, it has 2 key shortcomings. First, machine learning is harder to implement in real-world settings where many EHR systems can only accept regression or point-based approaches [13,14]. Second, it has lower explainability due to its black box nature. Clinicians may not accept black box models due to various reasons such as lack of external validation and the involvement of complex mathematical computation. Sullivan et al [15] suggested that the multivariable mathematical models are relatively complex, and the calculation should be simplified to allow application of models even without a computer, making these complex statistical models useful to clinical practitioners. Churpek et al [4] also suggested that a simple and parsimonious model can be applied at the bedside and easily validated across different hospitals. Thus, point-based scoring models are more favored in the medical context and are still widely used in clinical decision making. However, as developing a scoring model is nontrivial, there is a need to automate the process of score generation to cater to the increasingly diversified patient population and large-scale EHRs.

To tackle these problems and systematically present a robust and generic method for developing risk-scoring models, we proposed AutoScore, an automatic clinical score generator, by combining machine learning and regression modeling. The proposed AutoScore framework can automatically generate parsimonious sparse-score risk models (ie, risk scores), which can be easily implemented and validated in clinical practice. In this study, we implemented our proposed AutoScore framework to build an actual risk-scoring model for inpatient mortality prediction.

## Methods

### AutoScore for Automatic Score Generation

**Figure 1:**
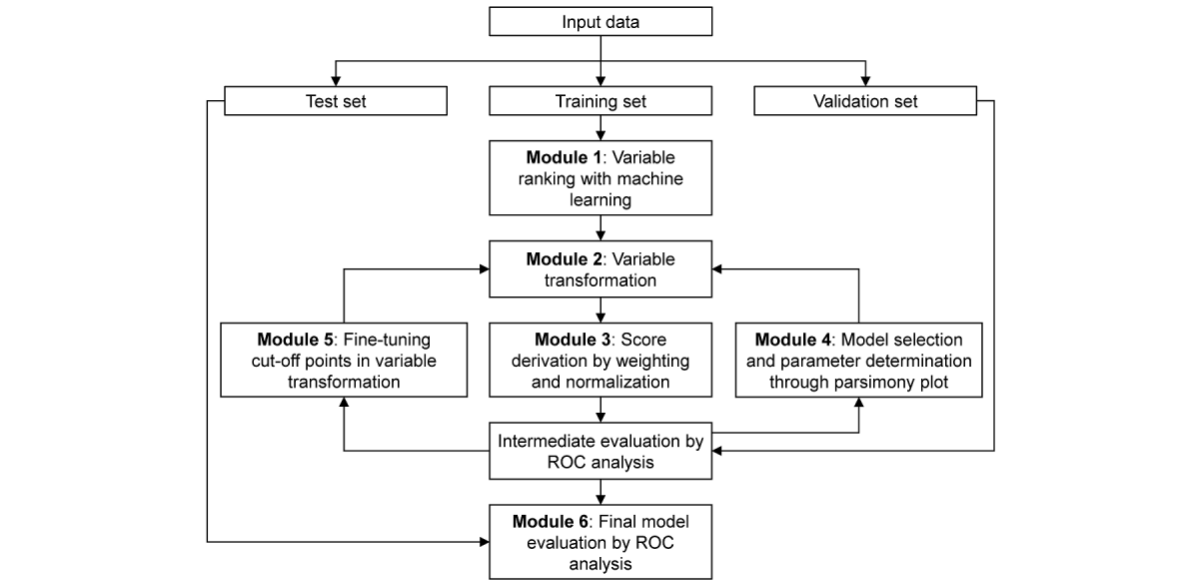
Flowchart of the AutoScore framework. ROC: receiver operating characteristic.

In this paper, we proposed the AutoScore, a novel framework for automating the development of a clinical scoring model for predefined outcomes and systematically presented its structure. AutoScore consists of 6 modules: variable ranking with machine learning, variable transformation, score derivation, model selection, domain knowledge–based score fine-tuning, and model evaluation. In our demonstration, the full data set was randomly split into a nonoverlapping training set (70%), validation set (10%; if downstream parameter tuning is needed), and test set (20%). The training set was used to derive the scores. The validation set was used for intermediate performance evaluation and parameter selection, which were elaborated in Module 4. The test set acted as an unseen data set and was used to generate the metrics of final model performance in Module 6. In real-world clinical applications, users can set up training, validation, and test sets accordingly instead of random splitting. Figure 1 illustrates the framework of AutoScore, and details of its 6 modules are elaborated as follows.

#### Module 1: Variable Ranking With Machine Learning

The first step in the AutoScore framework is variable ranking. We use random forest (RF) [16,17], an ensemble machine learning algorithm, to identify the top-ranking predictors for subsequent score generation. RF consists of multiple tree-structured classifiers (decision trees). Each of the trees is grown using a classification and regression tree [18] to maximum size, without pruning, and trained on a bootstrap sample and a random subset of all variables. Each tree sees only a subset of variables and part of the observations by resampling, which guarantees that the trees are decorrelated and, therefore, less prone to overfitting [19]. For the classification task, the Gini index is used to determine the optimal split. For each node 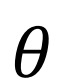 of a decision tree 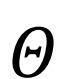, the Gini index can be defined as follows:

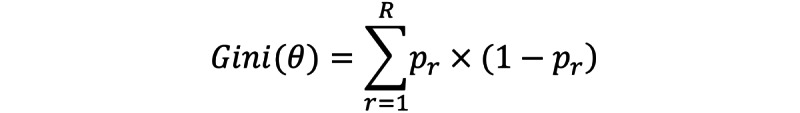



where *p_r_* refers to the fraction of training samples from the *r*^th^ class in the node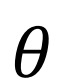 and *R=2* in binary classification. In addition to outcome prediction, RF ranks variables on the basis of their predictive importance [20]. The mean decrease impurity is the measurement of variable importance, calculated by the total decrease in node impurities from splitting on the variable. The importance measurement of a variable *X_m_* is the weighted total of impurity decreases* w(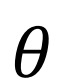)ΔGini(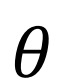)* for all nodes 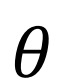
*,* averaged over all trees [21]:

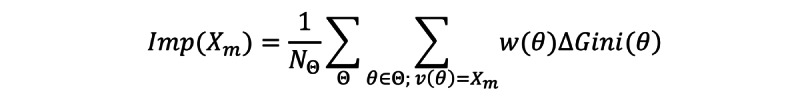



Where *w(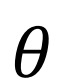)* is the proportional weight *N_
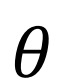
_/ N* of samples reaching node 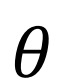, v(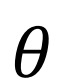) is the variable in the split of the node 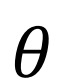, *ΔGini(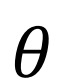)* is the total impurity decrease after the split of the node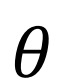; and *N_
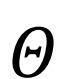
_* is the number of decision trees in the RF model. Then, *Imp(X_m_)* will be used for variable ranking for each *X_m_*.

An advantage of using RF as the variable ranker over other methods such as backward stepwise regression or LASSO is that as a nonparametric model, RF is able to rank variables on the basis of their nonlinear and heterogeneous effects. In the AutoScore framework, the final list of variables is decided by the ranking, in addition to the parameter *m*, which is the number of final selected variables. Parameter *m* can be chosen case by case in accordance with clinical preference, expert knowledge, or the needs of real-world applications. Moreover, an optimized number of variables can be determined through grid search and performance validation, which will be elaborated in Module 4.

#### Module 2: Variable Transformation

After variable selection, all selected variables are preprocessed for variable transformation, that is, continuous variables are converted into categorical variables. Creating categorical variables allows for the modeling of nonlinear effects. In AutoScore, the maximum number of categories (eg, *K*=5) for each variable is predefined to ensure its usability. For a categorical variable, if the original number of categories (*L*) exceeds the predefined maximum number (ie, *L*>*K*), several excess categories need to be combined, and *K'* is the number of categories of the transformed variable where *K'*≤*L*. Unlike categorical variables, to develop a point-based score, continuous variables will be stratified by specific quantiles into *K* categories (in our study, *K*=5). We set the quantiles as 0%, *k*_1_%, *k*_2_%, *k*_3_%, *k*_4_%, and 100%. The values of *k*_1_, *k*_2_, *k*_3_, and *k*_4_ can be set in accordance with the distributions of the variables. In our study, we set the default values as follows: *k*_1_=5, *k*_2_=20, *k*_3_=80, and *k*_4_=95, which were appropriate for most variables (such as common vital signs and laboratory test results), especially those with normal or near-normal distributions.

#### Module 3: Score Derivation by Weighting and Normalization

With the selected and transformed variables, we created a risk score to predict the outcome, in which each category of an individual variable is weighted and given an integer point. As the default setting, we used logistic regression for score weighting, with which the points can be easily interpreted.

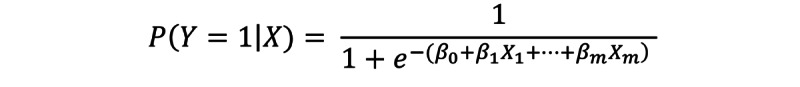



Where *β_0_* is the intercept, *β_1_*. . . *β_m_* are the coefficients for each category, *X_1_*. . . *X_m_* are the predictive variables, and *Y* is the binary outcome.

Multivariable logistic regression is performed to determine regression coefficients. On the basis of the results, the category of each variable with the lowest *β* coefficient is set as the reference. Next, multivariable logistic regression is performed again with adjusted reference categories to ensure that there are no negative coefficients. Subsequently, all coefficients *β* obtained from the second-round logistic regression are divided by the lowest *β* of all variables to ensure that all of the points are larger than one, that is, *β_new_ = β/β_lowest_*. The final weighted points for each category were rounded as *β_score_ = round(β_new_)*. With *β_score_*, we can obtain a scoring table where each category of a variable is given certain points. The total score is computed by summing up all points. To satisfy the need for specific clinical applications, we can set the ceiling value for the total score and normalize the score breakdowns, divided by a common denominator.

#### Module 4: Model Selection and Parameter Determination

The number of variables (*m*) is a critical parameter for controlling model complexity in the scoring model. A model is considered parsimonious when it is both sparse (using the least number of variables possible) and possesses a good prediction accuracy. To cope with the trade-off between accuracy and complexity, different parameter *m* will be examined on the validation set and a parsimony plot (ie, model performance vs complexity) will be plotted, to which the user can refer for deciding the trade-off in deriving the risk scores. The best parameter *m* is determined when *m* continues to increment and the prediction performance is no longer improving significantly, as shown in the parsimony plot. After confirming the parameter *m*, the final list of variables will be determined on the basis of the ranking obtained from Module 1. Modules 2 and 3 will be reimplemented to generate the initial scoring model.

#### Module 5: Fine-Tuning Cutoff Points in the Variable Transformation

Domain knowledge is essential in guiding risk model development. For continuous variables, the variable transformation (Module 2) is a data-driven process, in which domain knowledge is not integrated. In this module, the automatically generated cutoff values for each continuous variable can be fine-tuned by combining, rounding, and adjusting according to the standard clinical norm. The fine-tuning process endows the final risk scores with orderliness, professionality, and acceptability. After adjusting the cutoffs to convert continuous variables into categorical variables, Modules 2 and 3 will be implemented again to create an updated score table.

#### Module 6: Predictive Performance Evaluation

The performance of the score is evaluated on the basis of the receiver operating characteristic (ROC) analysis. The intermediate evaluation based on the validation set provides information for model optimization (eg, Modules 4 and 5). For the final model evaluation based on the unseen test set, the area under the ROC curve (AUC) acts as the primary metric. In addition, sensitivity, specificity, positive predictive value (PPV), and negative predictive value (NPV) are calculated under the optimal cutoffs, defined as the points nearest to the upper-left corner of the ROC curves. Performance metrics under different cutoffs are also compared to evaluate the predictive performance. In the demonstration, we included cutoffs, by which the sensitivity or specificity could reach about 95% to satisfy certain needs in clinical settings.

### Software Package

We have introduced all the 6 major modules of the AutoScore framework, with which clinical risk scores can be developed using specific patient cohorts and outcomes. We further created the AutoScore software suite [22] (Multimedia Appendices 1 and 2) under the R 3.5.3 (R Foundation) programming environment to demonstrate its capability and to facilitate its implementation and validation in other applications. Given a new data set, the AutoScore tool can be conveniently implemented to generate a point-based clinical scoring model to predict the outcome, with the minimum manual processes for data processing, parameter tuning, and model fine-tuning.

### Clinical Study Design

We conducted a retrospective analysis of data from the Beth Israel Deaconess Medical Center (BIDMC) to demonstrate the usability of our proposed AutoScore framework. BIDMC is a teaching hospital at the Harvard Medical School in Boston. It has 673 inpatient beds and receives about 55,000 emergency department visits annually. We aimed to implement AutoScore to automatically generate point-based scores for risk prediction of inpatient mortality and compared AutoScore-created scoring models with several baseline models.

#### Data Collection and Cohort

**Figure 2:**
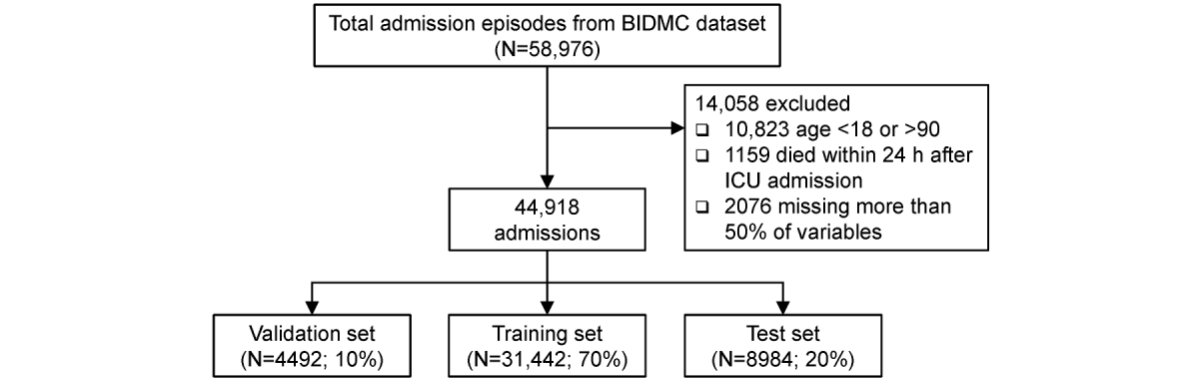
Flowchart of the study cohort formation. BIDMC: Beth Israel Deaconess Medical Center.

The BIDMC data set was obtained from the Medical Information Mart for Intensive Care III [23] database compiled by the Massachusetts Institute of Technology Laboratory for Computational Physiology. A total of 58,976 BIDMC admission encounters from 2001 to 2012 were recorded in this database. All inpatient encounters for which the patient aged 18 to 90 years were included in our study cohort. The admission episodes during which patients died within 24 hours after the intensive care unit (ICU) admission or missed more than 50% of the features were excluded. A flowchart of cohort formation is shown in Figure 2.

#### Variables and Clinical Outcome

The primary outcome in this study was inpatient mortality, defined as deaths that occurred during the hospital stay. In the BIDMC data set, we extracted patients’ first-day variables during their ICU stay. We previously demonstrated that demographic features, vital signs, and laboratory tests were highly related to inpatient mortality [24]. Similar results were also reported in other studies [25]. Thus, the predictor variables included age, sex, race, type of insurance, heart rate (beats/min), respiration rate (breaths/min), peripheral capillary oxygen saturation (SpO_2_; %), diastolic blood pressure (mm Hg), systolic blood pressure (mm Hg), mean arterial pressure (MAP; mm Hg), temperature (°C), bicarbonate (mmol/L), creatinine (μmol/L), potassium (mmol/L), sodium (mmol/L), hemoglobin (g/dL), glucose (mg/dL), blood urea nitrogen (BUN; mg/dL), platelet (thousand per microliter), lactate (mmol/L), anion gap (mEq/L), hematocrit (%), chloride (mEq/L), and white blood cells (thousand per microliter). As there were multiple sets of vital signs or laboratory data collected in the ICU, the mean values were used in this study.

#### Baseline Models Versus AutoScore

To evaluate the performance of AutoScore, we compared it with several standard predictive models. The first model was built with logistic regression by using all available variables from the training data set without variable selection. The second model was built using stepwise multivariable logistic regression [26]. It built a regression model with variable selection using the Akaike information criterion (AIC). Backward selection began with all the variables and removed the least significant one at each step following the declined AIC until none met the criterion. It penalized models with a large number of variables for a simple and parsimonious model. The third baseline model was built with LASSO [27], which is another popular method used in clinical modeling. It is a regression-based method that performs regularization for variable selection to improve both the predictive accuracy and interpretability of the statistical model. Its regularization rate was optimized through 10-fold cross-validation in our study. The last two baseline models were built using RF. We created both a full RF model using all available variables and an RF model using the AutoScore-selected variables. The parameters were selected according to the suggestions in previous literature [28,29], where *ntree*=100 and *mtry*=*m*^1/2^ (*ntree*: the number of trees grown; *mtry*: the number of variables randomly sampled as candidates at each split).

### Statistical Analysis and Model Evaluation

Data were analyzed using R 3.5.3 (R Foundation). The baseline characteristics of the data set are described. In the descriptive summaries, frequencies and percentages were reported for categorical variables, whereas means and SDs were reported for continuous variables. We compared patients with and without inpatient mortality using a two-tailed Student *t* test for continuous variables and the χ^2^ test for categorical variables. During the analysis, values of vital signs or laboratory tests were considered as outliers if they were beyond the normal range on the basis of domain knowledge. All detected outliers were set as missing values, which were subsequently imputed with the median values that were computed from the training set.

We compared the AutoScore-created scoring model with several baseline models to evaluate their predictive accuracy and interpretability. The test set was used to generate the metrics of model performance, and its bootstrapped samples were applied to calculate 95% CIs. Predictive accuracy was compared on the basis of ROC analysis and AUC values. Model interpretability was assessed by its complexity (eg, the number of variables included and the level of model nonlinearity) and its inherent explainability of the internal interaction. Model calibration was evaluated using the calibration belt plot test [30]. In addition, the distribution and observed mortality rate for each aggregated score were plotted for displaying its discriminative power.

## Results

### Baseline Characteristics of the Study Cohort

In this study, a total of 44,918 individual ICU admission episodes from the BIDMC data set were selected (Figure 2). Of all eligible episodes, 8.8% (3958/44,918) of the episodes had an outcome, that is, inpatient mortality. Summary baseline characteristics are shown in Table 1, and the distributions of other clinical continuous variables are shown in Table 2. In this cohort, the mean age was 62.5 (SD 16.5) years, 57.4% (25,788/44,918) were male, 84.9% (38,138/44,918) admissions were emergent, and the ethnic compositions were complex (31,889/44,918, 71.0% White; 4399/44,918, 9.8% African; 1625/44,918, 3.6% Hispanic; 1034/44,918, 2.3% Asian; and 5971/44,918, 13.3% others or unknown). We noticed that patients were admitted into different ICUs, which included Coronary Care Unit (CCU), Cardiac Surgery Recovery Unit (CSRU), Medical Intensive Care Unit (MICU), Surgical Intensive Care Unit (SICU), and Trauma Surgical Intensive Care Unit (TSICU). The average length of stay for all admission episodes was 4.19 (SD 6.11) days. Compared with the patients who survived to discharge, patients who died in hospitals were older, had a higher chance of emergency admission, had a longer length of stay, and a higher probability of being admitted to the MICU and paying by Medicare.

**Table 1:** Description of the study cohort (N=44,918).

Variables		All episodes (N=44,918)	Live discharged (n=40,960)	Inpatient mortality (n=3958)	*P* value	
Age (years), mean (SD)		62.5 (16.5)	62.0 (16.6)	68.5 (14.7)	<.001	
**Gender, n (** **%)**						.04
	Male	25,788 (57.4)	23,578 (57.6)	2210 (55.8)		
	Female	19,130 (42.6)	17,382 (42.4)	1748 (44.2)		
**Admission** **type, n (%)**						<.001
	Emergency	38,138 (84.9)	34,339 (83.8)	3799 (96.0)		
	Elective	6780 (15.1)	6621 (16.2)	159 (4.0)		
**Ethnicity, n** **(%)**						<.001
	White	31,889 (71.0)	29,148 (71.2)	2741 (69.3)		
	Hispanic	1625 (3.6)	1539 (3.8)	86 (2.2)		
	Asian	1034 (2.3)	933 (2.3)	101 (2.6)		
	African	4399 (9.8)	4110 (10.0)	289 (7.3)		
	Others or unknown	5971 (13.3)	5230 (12.8)	741 (18.7)		
**Insurance, n** **(%)**						<.001
	Government	1326 (3.0)	1258 (3.1)	68 (1.7)		
	Medicaid	4176 (9.3)	3896 (9.5)	280 (7.1)		
	Medicare	23,878 (53.2)	21,283 (52.0)	2595 (65.6)		
	Private	15,031 (33.5)	14,063 (34.3)	968 (24.5)		
	Self-pay	507 (1.1)	460 (1.1)	47 (1.2)		
**ICU^a^** **type, n** **(%)**						<.001
	CCU^b^	6445 (14.3)	5907 (14.4)	538 (13.6)		
	CSRU^c^	8284 (18.4)	8031 (19.6)	253 (6.4)		
	MICU^d^	17,490 (38.9)	15,420 (37.6)	2070 (52.3)		
	SICU^e^	7320 (16.3)	6649 (16.2)	671 (17.0)		
	TSICU^f^	5379 (12.0)	4953 (12.1)	426 (10.8)		
Length of stay (days), mean (SD)		4.19 (6.11)	3.87 (5.75)	7.57 (8.36)	<.001	

^a^ICU: intensive care unit.

^b^CCU: coronary care unit.

^c^CSRU: cardiac surgery recovery unit.

^d^MICU: medical intensive care unit.

^e^SICU: surgical intensive care unit.

^f^TSICU: trauma surgical intensive care unit.

**Table 2:** Distribution of clinical variables in the study cohort.

Variables	Values, median (IQR)
Age (years)	64.4 (51.9-75.9)
Heart rate (beats/min)	84.4 (74.5- 95.2)
Systolic blood pressure (mm Hg)	116.7 (107.1-129.5)
Diastolic blood pressure (mm Hg)	60 (53.7-67.4)
Mean arterial pressure (mm Hg)	76.9 (70.7-84.9)
Respiration rate (breaths/min)	18.0 (15.9-20.6)
Temperature (°C)	36.8 (36.5-37.2)
Peripheral capillary oxygen saturation (SpO_2_; %)	97.6 (96.2-98.7)
Glucose (mg/dL)	129.0 (111.3-154.3)
Anion gap (mEq/L)	13.5 (12-16)
Bicarbonate (mmol/L)	24.0 (21.5-26.5)
Creatinine (μmol/L)	0.95 (0.7-1.4)
Chloride (mEq/L)	105 (101.5-108)
Lactate (mmol/L)	1.8 (1.7-2.0)
Hemoglobin (g/dL)	10.9 (9.6-12.3)
Hematocrit (%)	32.3 (28.8-36.4)
Platelet (thousand per microliter)	208.5 (153.5-276.5)
Potassium (mmol/L)	4.2 (3.8-4.5)
Blood urea nitrogen (mg/dL)	18.0 (12.5-29.5)
Sodium (mmol/L)	138.5 (136-140.5)
White blood cells (thousand per microliter)	10.7 (8.0-14.3)

### Comparison of Selected Variables

**Figure 3:**
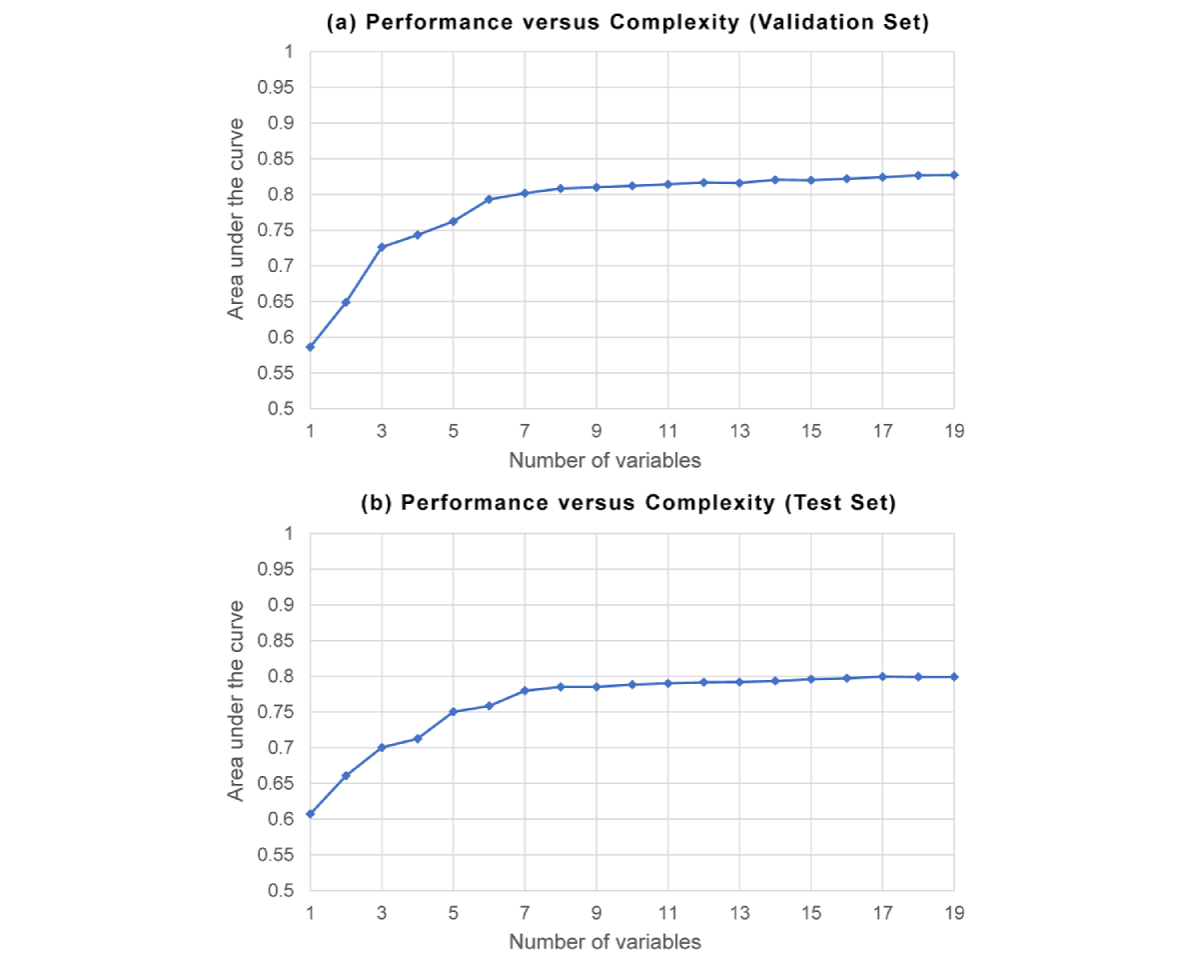
Model performance versus complexity for the implementation of the AutoScore on (a) the validation set and (b) the test set. The area under the curve reflects the discrimination performance, whereas the number of variables represents the complexity of the model.

Table 3 depicts the comparison of selected variables in the final model with different methods. The stepwise regression selected 22 variables, whereas the LASSO algorithm selected 17 variables after parameter tuning by 10-fold cross-validation. AutoScore selected a predefined number (*m*) of variables, and parameter *m* was optimized by a parsimony plot (ie, model performance vs complexity) on the validation set. As shown in part (a) of Figure 3, we chose 9 variables as the parsimonious choice as it achieved a good balance in the parsimony plot. When more variables were added to the scoring model, the performance was not markedly improved. Nine and 12 were selected as the number of variables in the demonstration. Users can also choose another parameter *m* if other restrictions or clinical preferences exist in real-life application scenarios. As seen from Table 3, the selected variables of AutoScore mostly coincided with those of the stepwise regression and LASSO. Notably, AutoScore generated a more parsimonious selection and sparse solution, catering to user preference and practical need.

**Table 3:** Selected variables by AutoScore and other baseline models.

Variables	Stepwise	LASSO	AutoScore (*m*=12)^a^	AutoScore (*m*=9)^a^
Age (years)	✔^b^	✔	✔	✔
Ethnicity	✔	✔	—^c^	—
Insurance	✔	✔	—	—
Gender	—	—	—	—
Heart rate	✔	✔	✔	✔
Systolic blood pressure	✔	✔	✔	✔
Diastolic blood pressure	✔	—	—	—
Mean arterial pressure	✔	✔	—	—
Respiration rate	✔	✔	✔	✔
Temperature	✔	✔	✔	✔
SpO_2_^d^	✔	✔	✔	✔
Glucose	✔	✔	✔	—
Anion gap	✔	—	—	—
Bicarbonate	✔	✔	✔	—
Creatinine	✔	—	—	—
Chloride	✔	✔	—	—
Hematocrit	✔	✔	—	—
Hemoglobin	✔	—	—	—
Lactate	✔	✔	✔	✔
Platelet	✔	✔	✔	✔
Potassium	✔	✔	—	—
BUN^e^	✔	—	✔	✔
Sodium	—	✔	—	—
White blood cells	✔	—	✔	—

^a^Parameter *m* is the number of variables included in the AutoScore model.

^b^Tick mark represents that this variable is included by the corresponding method.

^c^This variable is not included by the corresponding method.

^d^SpO_2_: peripheral capillary oxygen saturation.

^e^BUN: blood urea nitrogen.

### Scoring Models by AutoScore

**Figure 4:**
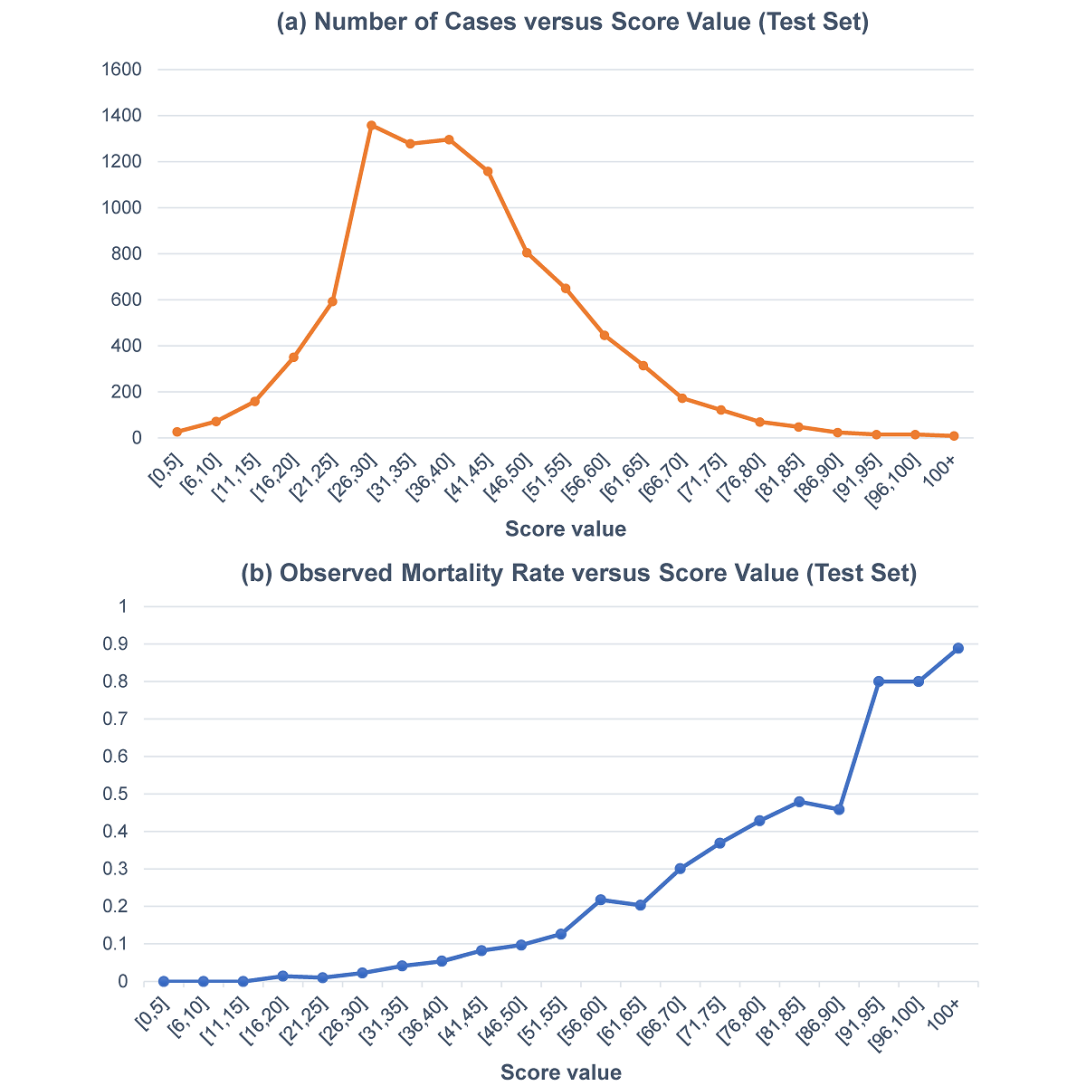
(a) Number of cases and (b) observed mortality rate, versus different score intervals obtained by the nine-variable AutoScore model.

The nine-variable AutoScore-created scoring model of inpatient mortality for the BIDMC data set is tabulated in Table 4. Age, heart rate, respiration rate, systolic blood pressure, SpO_2_, temperature, BUN, platelet, and lactate levels were selected into the final models. The final score summed up from 9 breakdowns ranged from 0 to 162. We used the test set to evaluate the property of this nine-variable point-based score. Part (a) of Figure 4 depicts the distribution of episodes at different score intervals, which is a near-normal distribution. Most patients had a risk score from 21 to 50, and very few patients had scores under 10 or above 80. As seen in part (b) of Figure 4, the observed mortality rate increased as our risk scores grew on the test set. The observed mortality rate was about 10% for a score of 50, whereas the mortality rate was over 50% for scores above 90. In terms of different breakdowns of the score, when age was lower than 30 years, its corresponding risk was the lowest; when it was higher than 85 years, the risk was the highest. Similarly, when the reported temperature was between 36.5°C and 37.5°C, the corresponding risk was the lowest, and when it was lower than 36°C, the risk was the highest. In addition, some variables, such as age, SpO_2_, and BUN, have larger score values, indicating more significant contributions to the risk.

**Table 4:** A nine-variable AutoScore-created scoring model for inpatient mortality.

Variables and interval^a^		Point
**Age (years)**		
	<30	0
	30-48	5
	48-78	14
	78-85	22
	≥85	24
**Heart rate (beats/min)**		
	<62	1
	62-72	0
	72-98	1
	98-112	8
	≥112	13
**Respiration rate (breaths/min)**		
	<12	3
	12-16	0
	16-22	4
	≥22	12
**Systolic blood pressure (mm Hg)**		
	<90	15
	90-100	8
	100-130	0
	130-150	1
	≥150	3
**Temperature (°C)**		
	<36	12
	36-36.5	3
	36.5-37.5	0
	37.5-38	5
	≥38	9
**SpO_2_^b^(%)**		
	<85	25
	85-90	13
	90-95	4
	≥95	0
**Platelet (thousand per microliter)**		
	<80	17
	80-150	3
	150-300	0
	300-450	3
	≥450	5
**Blood urea nitrogen (mg/dL)**		
	<7.5	0
	7.5-12	2
	12-35	9
	35-70	19
	≥70	23
**Lactate (mmol/L)**		
	<1	0
	1-2.5	2
	2.5-4	8
	≥4	21

^a^Interval (q_1_-q_2_) represents q_1_ ≤*x*<q_2_.

^b^SpO_2_: peripheral capillary oxygen saturation.

### Comparison of Predictive Performance

The results of mortality prediction, as assessed by the ROC analysis on the unseen test set, are reported in Table 5. The scoring models generated by AutoScore showed promising discriminatory capability in predicting inpatient mortality. The 12-variable AutoScore model achieved an AUC of 0.789 (95% CI 0.773-0.802) with a sensitivity of 71.7% (95% CI 68.5%-74.7%) and a specificity of 71.7% (95% CI 70.7%-72.7%) under the optimal threshold (score=130). When we compromised on accuracy for parsimony, the nine-variable AutoScore model achieved a slightly lower AUC of 0.780 (95% CI 0.764-0.798) with a sensitivity of 63.7% (95% CI 60.3%-67.1%) and a specificity of 77.2% (95% CI 76.3%-78.2%) under the optimal threshold (score=48). In comparison, the performance of the 24-variable full logistic regression, the 22-variable stepwise regression, the 17-variable LASSO models, the nine-variable RF model, and the 24-variable full RF model achieved AUC values of 0.778 (95% CI 0.760-0.795), 0.778 (95% CI 0.760-0.795), 0.772 (95% CI 0.755-0.790), 0.785 (95% CI 0.768-0.801), and 0.809 (95% CI 0.794-0.825), respectively. Table 5 presents the performance metrics that were calculated under different score cutoffs. Besides the optimal cutoffs, other cutoffs by which the sensitivity or specificity could reach approximately 95% were also evaluated.

**Table 5:** Performance of the AutoScore and other baseline models.

Methods, AUC^a^ (95% CI)		*m* ^b^	Threshold	Sensitivity (%), 95% CI	Specificity (%), 95% CI	PPV^c^ (%), 95% CI	NPV^d^ (%), 95% CI
**AutoScore (*m*^b^=9)**							
	0.780 (0.764-0.798)	9	48^e^	63.7 (60.3-67.1)	77.2 (76.3-78.2)	20.9 (19.8-22.0)	95.8 (95.4-96.1)
	N/A^f^	N/A	30^g^	95.7 (94.3-97.2)	25.1 (24.2-26.0)	10.8 (10.6-10.9)	98.4 (97.9-98.9)
	N/A	N/A	64^h^	28.8 (25.7-32.0)	95.5 (95.0-95.9)	37.6 (34.2-41.0)	93.4 (93.2-93.7)
**AutoScore (*m*^b^=12)**							
	0.789 (0.773-0.802)	12	130^e^	71.7 (68.5-74.7)	71.7 (70.7-72.7)	19.3 (18.4-20.1)	96.4 (96.0-96.8)
	N/A	N/A	95^g^	93.7 (92.0-95.3)	34.5 (33.4-35.6)	11.9 (11.6-12.1)	98.3 (97.8-98.7)
	N/A	N/A	180^h^	32.0 (28.8-35.3)	94.8 (94.3-95.2)	36.6 (33.4-39.9)	93.7 (93.4-94.0)
**Full logistic regression**							
	0.778 (0.760-0.795)	24	0.085^e^	68.6 (65.4-71.8)	72.8 (71.8-73.7)	19.2 (18.3-20.1)	96.1 (95.7-96.5)
	N/A	N/A	0.028^g^	95.2 (93.5-96.6)	25.3 (24.4-26.3)	10.7 (10.5-10.9)	98.3 (97.7-98.8)
	N/A	N/A	0.24^h^	27.9 (24.5-31.3)	95.1 (94.7-95.6)	35.0 (31.7-38.6)	93.3 (93.0-93.6)
**Stepwise regression**							
	0.778 (0.760-0.795)	22	0.096^e^	65.0 (61.6-68.5)	76.9 (76.0-77.8)	21.0 (19.9-22.0)	95.9 (95.5-96.3)
	N/A	N/A	0.028^g^	95.1 (93.5-96.5)	25.0 (24.1-26.1)	10.7 (10.5-10.9)	98.2 (97.6-98.7)
	N/A	N/A	0.24^h^	28.4 (25.1-31.7)	95.2 (94.7-95.6)	35.7 (32.2-39.1)	93.4 (93.1-93.7)
**LASSO^i^**							
	0.772 (0.755-0.790)	17	–2.47^e^	73.4 (70.2-76.4)	68.1 (67.1-69.2)	17.8 (17.0-18.6)	96.4 (96.0-96.8)
	N/A	N/A	–3.34^g^	95.2 (93.7-96.5)	25.1 (24.1-26.1)	10.7 (10.5-10.9)	98.2 (97.7-98.7)
	N/A	N/A	–1.27^h^	28.4 (25.2-31.8)	95.2 (94.7-95.7)	36.0 (32.6-39.5)	93.4 (93.1-93.7)
**Random forest(*m*^b^=9)^j^**							
	0.785 (0.768-0.801)	9	0.085^e^	74.2 (71.1-77.0)	69.4 (68.4-70.4)	18.6 (17.8-19.4)	96.6 (96.2-97.0)
	N/A	N/A	0.015^g^	94.2 (92.5-95.7)	30.1 (29.1-31.1)	11.3 (11.1-11.5)	98.2 (97.7-98.7)
	N/A	N/A	0.3^h^	30.5 (27.4-34.0)	94.8 (94.4-95.3)	35.7 (32.5-39.0)	93.5 (93.3-93.8)
**Full random forest**							
	0.809 (0.794-0.825)	24	0.115^e^	73.1 (69.9-76.2)	75.4 (74.5-76.3)	21.9 (20.9-22.9)	96.8 (96.4-97.1)
	N/A	N/A	0.025^g^	94.4 (92.8-95.9)	37.9 (36.9-38.9)	12.5 (12.3-12.8)	98.6 (98.2-99.0)
	N/A	N/A	0.285^h^	34.1 (30.6-37.5)	95.1 (94.6-95.5)	39.4 (36.2-42.9)	93.9 (93.6-94.2)

^a^AUC: the area under the ROC curve.

^b^Number of variables in the model.

^c^PPV: positive predictive value.

^d^NPV: negative predictive value.

^e^Optimal cutoff values, defined as the points nearest to the upper-left corner of the ROC curves.

^f^N/A: not applicable.

^g^Cutoff values by which the sensitivity could reach about 95%.

^h^Cutoff values by which the specificity could reach about 95%.

^i^LASSO: least absolute shrinkage and selection operator.

^j^AutoScore-based variable selection was implemented beforehand, where the same set of variables were selected as the AutoScore (*m*=9).

**Figure 5:**
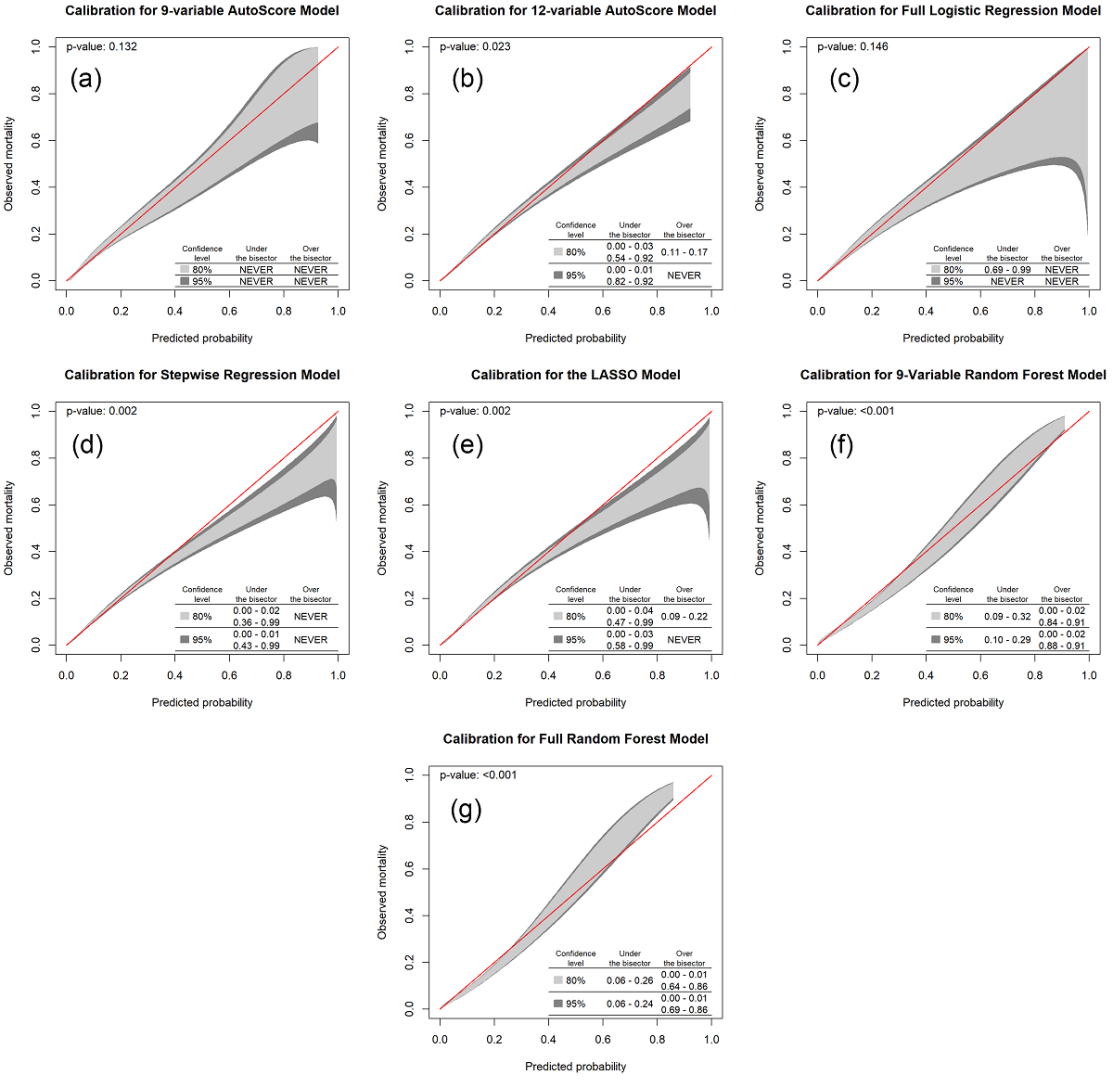
Calibration belts (at 80% and 95% confidence levels) for (a) a nine-variable AutoScore-created model, (b) a 12-variable AutoScore-created model, (c) a full logistic regression model, (d) a stepwise regression model, (e) the LASSO model, (f) a nine-variable random forest model, and (g) a full random forest model.

As illustrated in Figure 5, our nine-variable AutoScore model remained well calibrated, and all parts of the calibration belt showed a good fit under both 80% and 95% CIs. In comparison, other models displayed varying degrees of underestimation or overestimation. Two RF models performed the worst in the calibration test, followed by the stepwise regression and LASSO models. On the contrary, the AutoScore and logistic regression perform relatively well in terms of model calibration.

## Discussion

### Principal Findings

In this study, we developed AutoScore, a framework of automatic clinical score creation, and tested it in a large clinical data set. The scoring models generated by AutoScore were comparable with other standard methods (ie, logistic regression, stepwise regression, LASSO, RF model) in terms of predictive performance and model calibration. More importantly, the AutoScore-created scoring models showed superiority in interpretability and accessibility, as they were point-based scores with fewer variables used. In clinical practice, point-based scores have the advantage of easy implementation and, thus, can be widely utilized and validated in different circumstances and health care settings. The novelty of our study was the development of a generic, scalable, and robust methodology for automatically generating a point-based scoring model, which has been demonstrated by deriving an actual scoring model of inpatient mortality with a large benchmark EHR data set.

The proposed AutoScore has several advantages in creating risk prediction models. First, the machine learning–based variable ranking or selection can efficiently filter out redundant information. The importance of including variable selection in the development of predictive models has been demonstrated in many studies. In a study by Zhao et al [31], variable selection removed noninformative variables from the clinical predictive model. Bagherzadeh-Khiabani et al [32] demonstrated that the use of variable selection could improve the performance of clinical prediction models. Sanchez-Pinto et al [11] also provided evidence of modern tree-based methods of variable selection with better parsimony in large data sets. Liu et al [33] demonstrated that machine learning–based variable selection was promising for discovering a few relevant and significant variables in the prediction of adverse cardiac events. Second, the module of variable transformation could improve the fit of models. Several studies [34,35] have reported U-shaped nonlinearity between continuous variables and health-related outcomes. According to expert opinion, the value of vital signs or laboratory tests is usually considered as an abnormal value if it is beyond a healthy normal range. Besides, the categorization of continuous variables remains to be a dominant practice in epidemiological studies [36]. Discretizing features requires a smaller memory footprint, simplifies model interpretation, and can be applied directly by a human expert in routine care [37]. In addition, categorization creates a natural way to handle missing values, where the missing values can be treated as an extra category. This missing-indicator method has the appealing property that all available information can be used in the analyses [38]. Third, we use a parsimony plot (model performance vs complexity) to determine the appropriate number of variables (*m*), balancing the trade-off between performance and sparsity [39,40]. We value the model parsimony as the most desirable characteristic, as there is a real-world cost associated with mapping numerous variables, maintaining complex algorithms, and replicating it in different settings. This parsimony-driven parameter tuning process can be performed in an independent validation set (ie, 10% randomly selected samples from the entire data set in this study), as shown in Figure 3. It also shows a similar trend on the basis of the unseen test set, illustrating the effectiveness and consistency of parsimony-driven tuning for determining the number of necessary variables.

Furthermore, the scoring models created by the AutoScore framework are interpretable and clinically practical. The output of AutoScore is a point-based scoring model, based on addition, subtraction, and multiplication of a few sparse numbers, facilitating quick stratification without the need for a computing system. Doctors can easily understand how risk models make predictions in a transparent manner. Although numerous machine learning models, such as neural networks [41,42] and ensemble learning models [43,44], have been developed to complement traditional regression models, most of them are black boxes that do not explain their predictions in a way that humans can understand. In our study, the nine-variable RF model was performed as accurately as our nine-variable AutoScore (AUC 0.785 vs 0.780). However, it is challenging to explain the prediction made by the RF model, which consists of 100 different decision trees together. The lack of transparency of predictive models could lead to severe consequences in patient care. Vellido [12] suggested that these models with low explainability are unlikely to become part of routine clinical and health care practice as providing care is a highly sensitive task. Rudin [45] also suggested designing models that are inherently interpretable rather than explaining black box models and doubted the blind belief in the myth of the accuracy-interpretability trade-off.

### Relationship With Previous Work

Researchers have previously created several scoring models for predicting mortality, such as the Modified Early Warning Score [46], the VitalPAC Early Warning Score [47], and the Acute Physiology And Chronic Health Evaluation [48], mainly utilizing vital signs to predict mortality for hospitalized patients. However, they were designed by hand subjectively from expert opinions and domain knowledge, which hindered their generalization and dynamic evolution. Considering the disparate EHR systems among various health care settings, these scoring models may not work well because of the diversity among routinely collected information. As the characteristics of the population evolve, the adjustment and updating of risk scores are needed, which are time-consuming and inflexible [49]. In contrast, our AutoScore framework is adaptive and flexible; it can generate scoring models automatically, given an evolving EHR system. A user-friendly and easy-to-use R package of AutoScore [22] has been developed to facilitate the creation of scoring systems in diverse contexts, satisfying the increasing need for the development of specific predictive scores in various health care settings.

Similar to our AutoScore framework, Zhang et al [50] presented a tutorial on building a scoring system from several steps. However, the tutorial did not integrate some vital components such as variable ranking or selection and several crucial tuning processes inherently into the process of score generation. In comparison, our AutoScore framework includes all essential modules, driving the clinical continuum of 6 modules and realizing the automation. Although users may benefit from the built-in automation of AutoScore for developing a clinical score, domain knowledge is equally important in building the scoring models, as suggested in many studies [10,51]. In AutoScore, domain knowledge can be involved in 2 ways: (1) the variable can be preselected by expert opinion before implementing the AutoScore and (2) domain knowledge can be used to fine-tune the risk scores and determine clinically valid cutoff values in variable transformation.

### Future Research and Limitations

Although the proposed AutoScore framework is comprehensively and systematically presented, improvements can still be made. Each module of the AutoScore can be improved using advanced algorithms and enhanced methodologies. For example, in the module of variable ranking, various established machine learning methods can potentially be integrated into the AutoScore framework. In variable transformation, the means of categorization may be customized according to its distribution, provided a handful of clinical variables such as SpO_2_ that may not be subject to a near-normal distribution. Furthermore, the application of AutoScore is not limited to its application to large-scale EHR data [24,52]. AutoScore can be readily implemented in small-scale observational cohort studies. Beyond health care applications, AutoScore is potentially applicable to other high-stakes prediction applications such as criminal justice and finance, where highly interpretable predictive models are needed.

This study has several limitations. First, the data set used in this study was on the basis of EHR data with routinely collected vital and laboratory test variables. Some relevant variables were not available in this analysis. For example, health utilization, such as intubation and resuscitation, has been proven to be predictive of overall mortality. Second, given the limitation in data availability, the clinical scores built with AutoScore in this study are not perfect for real-world implementation. This clinical study was primarily designed to demonstrate the effectiveness of the AutoScore framework in building risk scores. Third, this was a retrospective analysis. To further prove its clinical practicability, prospective validation of the scoring model is needed. Finally, this was the initial development of AutoScore, where only selected methods were integrated into the framework, leaving opportunities for further development with more sophisticated and state-of-the-art algorithms.

### Conclusions

We developed an easy-to-use, machine learning–based automatic clinical score generator, AutoScore, to conveniently build scoring models and demonstrated its usability with a clinical study on mortality prediction. Using a benchmark data set, we showed that the scoring models derived with the AutoScore framework achieved satisfactory predictive performance and proved its superiority over several conventional methods for risk model development. The AutoScore framework integrates both the advantage of machine learning in strong discriminative power and the merit of point-based scores in its excellent accessibility and interpretability. Our proposed AutoScore framework can be readily used to generate clinical scores in various medical applications, such as early warning systems and risk predictions of mortality, hospital readmissions, and adverse cardiac events. In the future, advanced machine learning algorithms and methodologies could improve individual modules of AutoScore and provide AutoScore with more robust predictive capability or broader applicability in various types of data.

## Appendix

Multimedia Appendix 1 AutoScore R package (version 0.1).

Multimedia Appendix 2 AutoScore R package (version 0.1) source codes.

## Abbreviations

AIC: Akaike information criterion
AUC: area under the curve
BIDMC: Beth Israel Deaconess Medical Center
BUN: blood urea nitrogen
CCU: coronary care unit
CSRU: cardiac surgery recovery unit
EHR: electronic health record
ICU: intensive care unit
LASSO: least absolute shrinkage and selection operator
MAP: mean arterial pressure
MICU: medical intensive care unit
NPV: negative predictive value
PPV: positive predictive value
RF: random forest
ROC: receiver operating characteristic
SICU: surgical intensive care unit
TSICU: trauma surgical intensive care unit
