# Supplementary material for: AutoScore: A Machine Learning–Based Automatic Clinical Score Generator and Its Application to Mortality Prediction Using Electronic Health Records
Source: JMIR Med Inform. 2020 Oct 21;8(10):e21798. doi: 10.2196/21798 (PMC7641783; doi:10.2196/21798)
Supplement: Multimedia Appendix 1 [file medinform_v8i10e21798_app1.zip › AutoScore/html/00Index.html]

R: A Machine Learning-Based Automatic Clinical Score Generator

# A Machine Learning-Based Automatic Clinical Score Generator

---

## Documentation for package ‘AutoScore’ version 0.1

- DESCRIPTION file.

## Help Pages

|  |  |
| --- | --- |
| AutoScore-package | A Machine Learning-Based Automatic Clinical Score Generator |
| AutoScore\_fine\_tuning | Pepline function: STEP (4): Fine-tune the score(AutoScore Module 5) |
| AutoScore\_insample | Direct Automatic Clinical Score Generation (using in-sample validation) |
| AutoScore\_outofsample | Direct Automatic Clinical Score Generation (using out-of-sample validation) |
| AutoScore\_parsimony | Pepline function: STEP (2):Select the best model with parsimony plot (AutoScore Modules 2+3+4) |
| AutoScore\_rank | Pepline function: STEP (1):Genrate variable ranking List by machine learning (AutoScore Module 1) |
| AutoScore\_testing | Pepline function: STEP (5): Final score evaluation (AutoScore Module 6) |
| AutoScore\_weighting | Pepline function: STEP (3): Generate initial score with Final Variable list (Rerun AutoScore Module 2+3) |
| Descriptive | Descriptive |
| MultiVariable | Generate tables for Multivariable Analysis |
| Preprocess | Preprocess Dataset |
| UniVariable | Univariable Analysis |
