# Supplementary material for: AutoScore: A Machine Learning–Based Automatic Clinical Score Generator and Its Application to Mortality Prediction Using Electronic Health Records
Source: JMIR Med Inform. 2020 Oct 21;8(10):e21798. doi: 10.2196/21798 (PMC7641783; doi:10.2196/21798)
Supplement: Multimedia Appendix 1 [file medinform_v8i10e21798_app1.zip › AutoScore/html/AutoScore-package.html]

R: A Machine Learning-Based Automatic Clinical Score Generator

|  |  |
| --- | --- |
| AutoScore-package {AutoScore} | R Documentation |

## A Machine Learning-Based Automatic Clinical Score Generator

### Description

AutoScore, a novel framework to automate the
development of a clinical scoring model for predefined outcomes. AutoScore consists of six modules: variable ranking with
machine learning, variable transformation, score derivation, model selection, domain
knowledge-based score fine-tuning, and performance evaluation. The details are described in the manuscript<http://dx.doi.org/10.2196/21798>. USers or clinicians could seamlessly generate parsimonious sparse-score risk models (i.e.,
risk scores), which can be easily implemented and validated in clinical practice. Also, it enables users to build transparent and straightforward clinical scores quickly. We hope to see its application in various medical case studies.

The five pepline function `AutoScore_rank()`,`AutoScore_parsimony()`,`AutoScore_weighting()`,`AutoScore_fine_tuning()` and `AutoScore_testing()` constitute the standard 5-step AutoScore-based Score generation process. This 5-step process could conbineboth automation and customization. Users only need to do some choices (eg. determine final variable according to the parsimony plot, or fine-tune the cut-offs). Please follow the Guidebook and build you own scores using your own data step-by-step.
STEP (1): Generate variable ranking List (AutoScore Module 1)
STEP (2): Select the best model with parsimony plot (AutoScore Modules 2+3+4)
STEP (3): Generate initial score with Final Variable list (Rerun AutoScore Module 2+3)
STEP (4): Fine-tune the score- Revise CutVec with domain knowledge to update scoring table (AutoScore Module 5)
STEP (5): Final score evaluation (AutoScore Module 6)

We also provide some direct function `AutoScore_insample()`,`AutoScore_outofsample()`. These two functions can take input data and predefined parameter m (number of variables we need) to generate the scoring table and evaluate the model by the in-sample or out-of-sample validation. Although it's more automatic, it's also less flexible for routine use. We would recommend a 5-step standard procedure for generating your scoring model.

We also have other attached or related functions in the package, which are optional to use. They include `Preprocess()` for preprocessing you dataset(your own preprocessing is also okay),`Descriptive` for generating the descriptive table(table one) of your dataset, `UniVariable` for creating the table of Univariable analysis for your dataset,`MultiVariable` for generating the table of Multivariable analysis for your dataset. These functions are handy in building predictive models, especially for clinical manuscript formation.

### Details

Package: AutoScore
Type: Package
Title: A Machine Learning-Based Automatic Clinical Score Generator
Version: 0.1
Date: 2020-06-26
Author: Xie Feng<xief@u.duke.nus.edu>,Justin, Liu Nan<liu.nan@duke-nus.edu.sg>
Maintainer: Xie Feng<xief@u.duke.nus.edu>
License: GPL (>= 2)
Encoding: UTF-8
LazyData: true
RoxygenNote: 7.0.2
Imports:
tableone,
pROC,
randomForest,
ggplot2,
caret

### Examples

```
## Load R packages(besides AutoScore package)
#library(caret)
library(AutoScore)
library(pROC)
library(randomForest)
library(ggplot2)

## Load data (input data from csv or excel)
## can use sample data built in the package for the demo
df_AutoScore <- Sample_Data

##------------------------------------------------------------------
## Data preprocessing
## - Users are suggested to handle missing values, outliers, etc
## - Make Sure data are in good quality and reasonable distribution before entering our AutoScore Pipeline
## - "Preprocess" built-in function may help in missing value imputation
##
## Prepare TrainSet, ValidationSet, and Testset
## - Users need to define datasets to train, validate, and test model
## - Users can also use codes below to split their dataset into Train/validation/test(7:1:2)
##
## Other requirements for Data input
## - Independent variables (X) can be numeric (class: num / int) or categorical (class: factor/logic).
## - Categories/levels for each factor should be less than 10
## - Variables of character class (in R environment) were not accepted. Please transfer them into categorical variables first before going on with this codebook
## - Dependent variable (Y) should be binary, and its name should be changed to "label" (Can use codes below to do it.)
##-------------------------------------------------------------------

## Change name of Dependent variable (Y)/Outcome to "label" before going on with this codebook
names(df_AutoScore)[names(df_AutoScore)=="Mortality_inpatient"]<-"label"

## Data Splitting
set.seed(4)
Testindex <- sample((1:10000), 2000)
Validateindex <- sample((1:10000)[(1:10000) 

TrainSet <- df_AutoScore[-c(Validateindex, Testindex),]
TestSet <- df_AutoScore[Testindex,]
ValidationSet <- df_AutoScore[Validateindex,]

## Data displaying
head(TrainSet)
head(ValidationSet)
head(TestSet)

##-------------------------------------------------------------------
## Run AutoScore to build clinical scores: 5-step standard process
##-------------------------------------------------------------------

## STEP (1): Genrate variable ranking List (AutoScore Module 1)
## - ntree: Number of trees in random forest algorithm, default:100
Ranking <- AutoScore_rank(TrainSet, ntree=100)

## STEP (2): Select the best model with parsimony plot (AutoScore Modules 2+3+4)
## - nmin: Minimum number of selected variables, default:1
## - nmax: Maximum number of selected variables, default:20
## - probs: Predefine quantiles to convert continuous variables to categorical, default:(0, 0.05, 0.2, 0.8, 0.95, 1)
AUC <- AutoScore_parsimony(TrainSet, ValidationSet, rank=Ranking, nmin=1, nmax=20, probs=c(0, 0.05, 0.2, 0.8, 0.95, 1))

## Determine final num_var for predictive modeling
## -- decided with the parsimony plot from STEP(2)
num_var <- 6
FinalVariable <- names(Ranking[1:num_var])

## STEP (3): Generate initial score with Final Variable list (Rerun AutoScore Module 2+3)
## - MaxScore: Predefined cap of final score, e.g. 100
CutVec <- AutoScore_weighting(TrainSet, ValidationSet, FinalVariable, MaxScore=100, probs=c(0, 0.05, 0.2, 0.8, 0.95, 1))

## STEP (4): Fine-tune the score
## - Revise CutVec with domain knowledge to update scoring table (AutoScore Module 5)
## - Rerun AutoScore Modules 2+3
## - User can choose any cut-off values/any number of categories
CutVec$tempc_mean <- c(36, 36.5, 37.3, 38)
CutVec$platelet_min <- c(60, 120, 280, 400)
CutVec$lactate_max <- c(1, 1.7, 2.8, 5.7)
CutVec$resprate_mean <- c(13, 16, 21, 26)
CutVec$spo2_mean <- c(95, 99)
ScoringTable <- AutoScore_fine_tuning(TrainSet, ValidationSet, FinalVariable, CutVec, MaxScore=100)

## STEP (5): Final score evaluation (AutoScore Module 6)
AutoScore_testing(TestSet, FinalVariable, CutVec, ScoringTable)


##-------------------------------------------------------------------
## Run AutoScore to build clinical scores: direct function usage
##-------------------------------------------------------------------

# preprocessing
# df_AutoScore<-Preprocess(Sample_Data,outcome = "Mortality_inpatient")

## This function is used to generate scoring model based on a dataset and predefined number of variables. And it uses all sample for performance evaluation, which is good for some studied with small sample size.
AutoScore_insample(df_AutoScore, m = 6, MaxScore = 150, probs = c(0, 0.05, 0.2, 0.8, 0.95, 1))

## This function is used to generate scoring model based on a dataset and predefined number of variables. And it uses testing data randomly selected from the original dataset for performance evaluation, which is good for some studied with relatively big sample size.
AutoScore_outofsample(df_AutoScore, m = 6 , Percentage_test = 0.2, MaxScore = 150, probs = c(0, 0.05, 0.2, 0.8, 0.95, 1))


##-------------------------------------------------------------------
## Other analysis direct function usage
##-------------------------------------------------------------------

## Descriptive Analysis and generate the result table
Descriptive(df_AutoScore)

## Univariable Analysis and generate the result table
UniTable<-UniVariable(df_AutoScore)

## Multivariable Analysis and generate the result table
MultiTable<-MultiVariable(df_AutoScore)
```

---

[Package *AutoScore* version 0.1 Index]
