# Supplementary material for: AutoScore: A Machine Learning–Based Automatic Clinical Score Generator and Its Application to Mortality Prediction Using Electronic Health Records
Source: JMIR Med Inform. 2020 Oct 21;8(10):e21798. doi: 10.2196/21798 (PMC7641783; doi:10.2196/21798)
Supplement: Multimedia Appendix 1 [file medinform_v8i10e21798_app1.zip › AutoScore/html/AutoScore_fine_tuning.html]

R: Pepline function: STEP (4): Fine-tune the score(AutoScore...

|  |  |
| --- | --- |
| AutoScore\_fine\_tuning {AutoScore} | R Documentation |

## Pepline function: STEP (4): Fine-tune the score(AutoScore Module 5)

### Description

STEP (4): Fine-tune the score
- Revise CutVec with domain knowledge to update scoring table (AutoScore Module 5)
- Rerun AutoScore Modules 2+3
- User can choose any cut-off values/any number of categories

### Usage

```
AutoScore_fine_tuning(TrainSet, ValidationSet, FinalVariable, CutVec, MaxScore=100)
```

### Arguments

|  |  |
| --- | --- |
| `TrainSet` | a dataframe that is Training set |
| `ValidationSet` | a dataframe that is Validation Set |
| `FinalVariable` | Final list of variables, generated from last step |
| `MaxScore` | Predefined cap of final score, e.g. 100 |
| `CutVec` | Generated from STEP(3) `AutoScore_weighting().Please follow the guidebook` |

### Value

Generated final table of scoring model for downstream testing process[STEP (5)]

### Examples

```
ScoringTable <- AutoScore_fine_tuning(TrainSet, ValidationSet, FinalVariable, CutVec, MaxScore=100)
```

---

[Package *AutoScore* version 0.1 Index]
