# Supplementary material for: AutoScore: A Machine Learning–Based Automatic Clinical Score Generator and Its Application to Mortality Prediction Using Electronic Health Records
Source: JMIR Med Inform. 2020 Oct 21;8(10):e21798. doi: 10.2196/21798 (PMC7641783; doi:10.2196/21798)
Supplement: Multimedia Appendix 1 [file medinform_v8i10e21798_app1.zip › AutoScore/html/AutoScore_insample.html]

R: Direct Automatic Clinical Score Generation (using in-sample...

|  |  |
| --- | --- |
| AutoScore\_insample {AutoScore} | R Documentation |

## Direct Automatic Clinical Score Generation (using in-sample validation)

### Description

This function is used to generate scoring model based on a dataset and predefined number of variables.
And it uses all sample for performance evaluation, which is good for some studied with small sample size.

### Usage

```
AutoScore_insample(data, m, MaxScore = 100, probs = c(0, 0.05, 0.2, 0.8, 0.95, 1))
```

### Arguments

|  |  |
| --- | --- |
| `data` | a dataframe that contains data to be analysed |
| `m` | Predefined number of variables to be selected |
| `MaxScore` | Predefined cap of final score, e.g. 100 |
| `probs` | Predefine quantiles to convert continuous variables to categorical, default:(0, 0.05, 0.2, 0.8, 0.95, 1) |

### Value

List of parameters generated by model: including list of variable selected, scoring table and performance evaluation (in-sample)

### Examples

```
AutoScore_insample(data=Sample_Data, m=8))
```

---

[Package *AutoScore* version 0.1 Index]
