# Supplementary material for: AutoScore: A Machine Learning–Based Automatic Clinical Score Generator and Its Application to Mortality Prediction Using Electronic Health Records
Source: JMIR Med Inform. 2020 Oct 21;8(10):e21798. doi: 10.2196/21798 (PMC7641783; doi:10.2196/21798)
Supplement: Multimedia Appendix 1 [file medinform_v8i10e21798_app1.zip › AutoScore/html/AutoScore_outofsample.html]

R: Direct Automatic Clinical Score Generation (using...

|  |  |
| --- | --- |
| AutoScore\_outofsample {AutoScore} | R Documentation |

## Direct Automatic Clinical Score Generation (using out-of-sample validation)

### Description

This function is used to generate scoring model based on a dataset and predefined number of variables.
And it uses testing data randomly selected from the original dataset for performance evaluation, which is good for some studied with relatively big sample size.(if sample size is small, please use `AutoScore_insample()`)

### Usage

```
AutoScore_outofsample(Dataset, m = 8 , Percentage_test = 0.2, MaxScore = 100, probs = c(0, 0.05, 0.2, 0.8, 0.95, 1))
```

### Arguments

|  |  |
| --- | --- |
| `Dataset` | a dataframe that contains data to be analysed |
| `m` | Predefined number of variables to be selected |
| `Percentage_test` | percentage of samples used for testing(out-of-sample validation), e.g. 0.2 |
| `MaxScore` | Predefined cap of final score, e.g. 100 |
| `probs` | Predefine quantiles to convert continuous variables to categorical, default:(0, 0.05, 0.2, 0.8, 0.95, 1) |

### Value

List of parameters generated by model: including list of variable selected, scoring table and performance evaluation (out-of-sample)

### Examples

```
AutoScore_outofsample(Sample_Data, m=8))
```

---

[Package *AutoScore* version 0.1 Index]
