# Supplementary material for: AutoScore: A Machine Learning–Based Automatic Clinical Score Generator and Its Application to Mortality Prediction Using Electronic Health Records
Source: JMIR Med Inform. 2020 Oct 21;8(10):e21798. doi: 10.2196/21798 (PMC7641783; doi:10.2196/21798)
Supplement: Multimedia Appendix 1 [file medinform_v8i10e21798_app1.zip › AutoScore/html/AutoScore_parsimony.html]

R: Pepline function: STEP (2):Select the best model with...

|  |  |
| --- | --- |
| AutoScore\_parsimony {AutoScore} | R Documentation |

## Pepline function: STEP (2):Select the best model with parsimony plot (AutoScore Modules 2+3+4)

### Description

STEP (2):Select the best model with parsimony plot (AutoScore Modules 2+3+4)

### Usage

```
AutoScore_parsimony(TrainSet, ValidationSet, rank=Ranking, nmin=1, nmax=20, probs=c(0, 0.05, 0.2, 0.8, 0.95, 1))
```

### Arguments

|  |  |
| --- | --- |
| `TrainSet` | a dataframe that is Training set |
| `ValidationSet` | a dataframe that is Validation Set |
| `rank` | the result generated from STEP (1) |
| `nmin` | Minimum number of selected variables, default:1 |
| `nmax` | Maximum number of selected variables, default:20 |
| `probs` | Predefine quantiles to convert continuous variables to categorical, default:(0, 0.05, 0.2, 0.8, 0.95, 1) |

### Value

List of AUC value for different parameter m and parsimony plot

### Examples

```
AUC <- AutoScore_parsimony(TrainSet, ValidationSet, rank=Ranking, nmin=1, nmax=20, probs=c(0, 0.05, 0.2, 0.8, 0.95, 1))
```

---

[Package *AutoScore* version 0.1 Index]
