# Supplementary material for: AutoScore: A Machine Learning–Based Automatic Clinical Score Generator and Its Application to Mortality Prediction Using Electronic Health Records
Source: JMIR Med Inform. 2020 Oct 21;8(10):e21798. doi: 10.2196/21798 (PMC7641783; doi:10.2196/21798)
Supplement: Multimedia Appendix 1 [file medinform_v8i10e21798_app1.zip › AutoScore/html/AutoScore_rank.html]

R: Pepline function: STEP (1):Genrate variable ranking List by...

|  |  |
| --- | --- |
| AutoScore\_rank {AutoScore} | R Documentation |

## Pepline function: STEP (1):Genrate variable ranking List by machine learning (AutoScore Module 1)

### Description

STEP (1): Genrate variable ranking List (AutoScore Module 1)

### Usage

```
AutoScore_rank(TrainSet, ntree=100)
```

### Arguments

|  |  |
| --- | --- |
| `TrainSet` | a dataframe that contains data to be analysed, for training |
| `ntree` | Number of trees in random forest algorithm, default:100 |

### Value

List of variables and its ranking generated by machine learning(random forest)

### Examples

```
Ranking <- AutoScore_rank(TrainSet, ntree=200)
```

---

[Package *AutoScore* version 0.1 Index]
