# Supplementary material for: AutoScore: A Machine Learning–Based Automatic Clinical Score Generator and Its Application to Mortality Prediction Using Electronic Health Records
Source: JMIR Med Inform. 2020 Oct 21;8(10):e21798. doi: 10.2196/21798 (PMC7641783; doi:10.2196/21798)
Supplement: Multimedia Appendix 1 [file medinform_v8i10e21798_app1.zip › AutoScore/html/AutoScore_testing.html]

R: Pepline function: STEP (5): Final score evaluation (AutoScore...

|  |  |
| --- | --- |
| AutoScore\_testing {AutoScore} | R Documentation |

## Pepline function: STEP (5): Final score evaluation (AutoScore Module 6)

### Usage

```
AutoScore_testing(TestSet, FinalVariable, CutVec, ScoringTable)
```

### Arguments

|  |  |
| --- | --- |
| `TestSet` | a dataframe that is Testing set |
| `FinalVariable` | Final list of variables, generated from last step |
| `ScoringTable` | Generated from STEP(4) `AutoScore_fine_tuning`.Please follow the guidebook |
| `CutVec` | Generated from STEP(3) `AutoScore_weighting().Please follow the guidebook` |

No return value. All performance evaluation would be printed out.
STEP (5): Final score evaluation (AutoScore Module 6)
AutoScore\_testing(TestSet, FinalVariable, CutVec, ScoringTable)

---

[Package *AutoScore* version 0.1 Index]
