# Supplementary material for: AutoScore: A Machine Learning–Based Automatic Clinical Score Generator and Its Application to Mortality Prediction Using Electronic Health Records
Source: JMIR Med Inform. 2020 Oct 21;8(10):e21798. doi: 10.2196/21798 (PMC7641783; doi:10.2196/21798)
Supplement: Multimedia Appendix 1 [file medinform_v8i10e21798_app1.zip › AutoScore/html/AutoScore_weighting.html]

R: Pepline function: STEP (3): Generate initial score with Final...

|  |  |
| --- | --- |
| AutoScore\_weighting {AutoScore} | R Documentation |

## Pepline function: STEP (3): Generate initial score with Final Variable list (Rerun AutoScore Module 2+3)

### Description

STEP (3): Generate initial score with Final Variable list (Rerun AutoScore Module 2+3)

### Usage

```
AutoScore_weighting(TrainSet, ValidationSet, FinalVariable, MaxScore=100, probs=c(0, 0.05, 0.2, 0.8, 0.95, 1)
```

### Arguments

|  |  |
| --- | --- |
| `TrainSet` | a dataframe that is Training set |
| `ValidationSet` | a dataframe that is Validation Set |
| `FinalVariable` | Final list of variables, generated from last step |
| `MaxScore` | Predefined cap of final score, e.g. 100 |
| `probs` | Predefine quantiles to convert continuous variables to categorical, default:(0, 0.05, 0.2, 0.8, 0.95, 1) |

### Value

Generated `CutVec` for downstream fine-tuning process[STEP (4)]

### Examples

```
CutVec <- AutoScore_weighting(TrainSet, ValidationSet, FinalVariable, MaxScore=100, probs=c(0, 0.05, 0.2, 0.8, 0.95, 1))
```

---

[Package *AutoScore* version 0.1 Index]
