# Supplementary material for: AutoScore: A Machine Learning–Based Automatic Clinical Score Generator and Its Application to Mortality Prediction Using Electronic Health Records
Source: JMIR Med Inform. 2020 Oct 21;8(10):e21798. doi: 10.2196/21798 (PMC7641783; doi:10.2196/21798)
Supplement: Multimedia Appendix 1 [file medinform_v8i10e21798_app1.zip › AutoScore/html/Descriptive.html]

R: Descriptive

|  |  |
| --- | --- |
| Descriptive {AutoScore} | R Documentation |

## Descriptive

### Description

Table one

### Usage

```
Descriptive(x)
```

### Arguments

|  |  |
| --- | --- |
| `x` | dataset after preprocessing |

### Value

print Descriptive table for your data, straified by your label(outcome)

### Examples

```
Descriptive(data)
```

---

[Package *AutoScore* version 0.1 Index]
