# Supplementary material for: AutoScore: A Machine Learning–Based Automatic Clinical Score Generator and Its Application to Mortality Prediction Using Electronic Health Records
Source: JMIR Med Inform. 2020 Oct 21;8(10):e21798. doi: 10.2196/21798 (PMC7641783; doi:10.2196/21798)
Supplement: Multimedia Appendix 1 [file medinform_v8i10e21798_app1.zip › AutoScore/html/MultiVariable.html]

R: Generate tables for Multivariable Analysis

|  |  |
| --- | --- |
| MultiVariable {AutoScore} | R Documentation |

## Generate tables for Multivariable Analysis

### Description

Generate tables for Multivariable Analysis

### Usage

```
MultiVariable(data)
```

### Arguments

|  |  |
| --- | --- |
| `data` | standard data frame |

### Value

Dataframe of results of multivariable analysis

### Examples

```
MultiVariable(data)
```

---

[Package *AutoScore* version 0.1 Index]
