# Supplementary material for: AutoScore: A Machine Learning–Based Automatic Clinical Score Generator and Its Application to Mortality Prediction Using Electronic Health Records
Source: JMIR Med Inform. 2020 Oct 21;8(10):e21798. doi: 10.2196/21798 (PMC7641783; doi:10.2196/21798)
Supplement: Multimedia Appendix 1 [file medinform_v8i10e21798_app1.zip › AutoScore/html/Preprocess.html]

R: Preprocess Dataset

|  |  |
| --- | --- |
| Preprocess {AutoScore} | R Documentation |

## Preprocess Dataset

### Description

Preprocess Dataset

### Usage

```
Preprocess(data, outcome)
```

### Arguments

|  |  |
| --- | --- |
| `data` | The data which will be the source for the dataset |
| `outcome` | The column within the dataset that determines the outcome |

### Value

The preprocessed dataset

### Examples

```
data <- Preprocess(testdf1_mimic, outcome = "label")
```

---

[Package *AutoScore* version 0.1 Index]
